# Single-nucleotide polymorphism rs1058205 of *KLK3* is associated with the risk of prostate cancer: A case–control study of Han Chinese men in Northeast China

**DOI:** 10.1097/MD.0000000000006280

**Published:** 2017-03-10

**Authors:** Chen Chen, Zhongqiu Xin

**Affiliations:** a Department of Urology; b Ultrasound Room, Daqing Oilfield General Hospital, Daqing, China.

**Keywords:** Han Chinese, prostate cancer, prostate-specific antigen, rs1058205, single-nucleotide polymorphism

## Abstract

**Background:**

Prostate cancer (PCa) is a serious public health concern for men worldwide. However, the risk factors for PCa remain largely unclear. Aim of this study was to investigate statistical associations between the risk of prostate cancer and the rs1058205 single-nucleotide polymorphism (SNP) of the KLK3 gene, which encodes the prostate specific antigen (PSA), in a case-control study of Han Chinese men in Northeast China.

**Methods:**

Using a high-resolution melting curve genotyping method, we determined the genotype and allele distributions of rs1058205 in 2 groups of Han Chinese men, consisting of 268 PCa patients and 298 healthy control subjects. Logistic regression was used to evaluate associations between rs1058205 genotypes and the risk of PCa. Tumor staging and Gleason score were included in a stratified analysis of PCa risk.

**Results:**

The frequency of the TC genotype of rs1058205 in the PCa group was significantly lower than that in the control group (*P* = 0.049). The serum PSA level in participants with the TC genotype was significantly lower than that of the TT and CC genotypes in both the PCa and control groups (*P* < 0.010 for both). The TT genotype was associated with PCa, both with and without adjustment for age (*P* < 0.010 and *P* = 0.047, respectively). The TT genotype was also associated with the moderate- and high-risk PCa categories (*P* = 0.007 and 0.027, respectively).

**Conclusion:**

The TT genotype may represent a useful biomarker for identifying high risk of PCa and as a postoperative prognosticator in Chinese PCa patients.

## 1 INTRODUCTION

Prostate cancer (PCa) is the most common cancer among men in developing countries.^[1]^ It is also the fifth most common cause of cancer-related death in men worldwide.^[2]^ The incidence of PCa in China is lower than that in North America, Europe, Australia, and New Zealand.^[1,2]^ However, although differences in the rates of PCa exist between men in urban and rural areas in China, the overall incidence of PCa is increasing, and become most common type of cancer among urban Chinese men,^[3,4]^ which highlights the need for improved screening methods for PCa to reduce cancer-related mortality in China.

Although no clear understanding exists regarding the environmental risk factors for PCa, such as pollutants, diet, and behavior, a wealth of evidence suggests that genetic factors are associated with PCa susceptibility. Twin studies in Scandinavian countries have indicated that 42% of the variation in PCa risk may be attributed to genetics, which is higher than that reported for other highly prevalent types of cancer.^[5]^ An international study found that approximately 30% of the estimated heritability of PCa can be attributed to single-nucleotide polymorphisms (SNPs).^[6]^ In a recent multinational study of 25 PCa susceptibility loci, the PRACTICAL Consortium concluded that risk profiling based on SNP analyses can identify men of European ancestry with a substantially increased risk of PCa.^[7]^ It is, however, unclear whether such screening would be effective for Chinese men.

For many years, the prostate-specific antigen (PSA), which plays an important role in sperm motility, has been used as a biomarker for PCa screening. The PSA protein is encoded by the kallikrein 3 (*KLK3*) gene, and the expression of PSA is mediated by the androgen receptor in both healthy and cancerous prostate tissues.^[8]^ Evidence from in vitro studies suggests that PSA is activated by kH2, a protein that is encoded by the closely related gene, kallikrein 2 (*KLK2*).^[9]^ PSA is also involved in the proteolytic breakdown of the extracellular matrix in PCa tumorigenesis, which contributes to tumor invasion and metastasis,^[10]^ and high serum PSA correlates with mutations in p53 and the overexpression of the B-cell lymphoma 2 protein, which inhibits apoptosis in tumor cells.^[11]^ These findings strongly suggest that PSA plays a role in the etiology of PCa.

Previous studies have shown that rs1058205, a tag SNP in the 3′ untranslated region of *KLK3* at the 19q13.33 locus, was associated with lower serum PSA levels in Swedish and African-American men,^[12,13]^ and that the TT genotype of rs1058205 was associated with reduced PCa aggressiveness in Caucasian American men based on Gleason scoring,^[13]^ which suggests that sequence variation at rs1058205 may protect against PCa in at least some populations. Zhang et al^[14]^ found no associations between any of the SNPs at the 19q13.33 locus, including rs1058205, and the risk of PCa in Han Chinese men in Beijing. However, their study is the only previous investigation performed that examined the relationship between rs1058205 and PSA/PCa among men in China. Therefore, whether rs1058205 is associated with PSA level or the risk of PCa in China, the world's most populous nation, has not been thoroughly investigated. We investigated whether rs1058205 was associated with the risk of PCa in Han Chinese men in Northeast China to evaluate possible associations between the rs1058205 genotypes and the risk of PCa within the Chinese subcontinent.

## 2 METHODS

### 2.1 Patients

Men who received a diagnosis of PCa at our institution in Daqing, Heilongjiang, China were enrolled in the PCa group between March 2012 and March 2014 for participation in our case–control observational study. Inclusion criteria for the PCa group included age >45 years, no history of other types of cancer, histopathologically confirmed PCa, and complete case record. The control group consisted of randomly selected, healthy men who had received routine physicals at our institution during the same period. Inclusion criteria for the control group included age >45 years, no family history of PCa, negative findings from digital rectal examination, and total serum PSA < 4 ng/mL. Written informed consent was obtained from all of the PCa patients and control subjects before their participation in our study. Our study was approved by the Ethics Committee of our institution and was performed in accordance with the Declaration of Helsinki with regard to ethical principles for research involving human subjects.

### 2.2 Clinical variables

Tumor aggressiveness was assessed based on Gleason score.^[15]^ Tumor staging was performed using both the Jewett-Whitmore^[16]^ and tumor–node–metastasis (TNM)^[17]^ staging systems. Pathological staging of PCa was defined according to the Jewett-Whitmore staging system as follows: A—incidentally discovered, B—localized, C—invasion to adjacent organs, or D—distant metastases or lymph node involvement. Jewett-Whitmore stages A and B were defined as nonmetastatic, and Jewett-Whitmore stages C and D were defined as metastatic. Risk categories were determined according to the National Comprehensive Cancer Network, Version 3, 2012.^[18]^ Low risk was defined as total serum PSA < 10 ng/mL, Gleason score ≤6, and a TNM stage ≤T2a. Moderate risk was defined as a total serum PSA level of 10 to 20 ng/mL, or Gleason score of 7, and or TNM stage of T2b. High risk was defined as total serum PSA >20 ng/mL, or Gleason score ≥8, and or TNM stage ≥T2c.

### 2.3 SNP genotyping

Blood was collected from each patient by venipuncture in Ethytlene Diamine Tetraacetic Acid-treated anticoagulant vacuum tubes, and 0.5 mL blood was centrifuged for 10 minutes at 3000 rpm. Genomic Deoxyribonucleic acid (DNA) was extracted from peripheral blood leukocytes in the buffy coat of centrifuged blood samples using a genomic DNA extraction kit (Boer Cheng Technology, Beijing, China). The concentration and purity of the purified DNA was spectrophotometrically assessed and confirmed by agarose gel electrophoresis and ethidium bromide staining before storage at 4 °C. Oligonucleotide primers were designed based on the sequence of the rs1058205 locus in the Genome 10k Ensemble database using the Oligo, Version 7.0, software (Molecular Biology Insights, Inc., Colorado Springs, CO), and primer synthesis was performed by Shenggong Biological Engineering (Shanghai, China).

The SNP genotyping was performed using a high-resolution melting (HRM) curve polymerase chain reaction (PCR) method, as described previously.^[19]^ The HRM forward (5′-CAACCCCGAGCACTCCC-3′) and HRM reverse (5′-CAAGTTCCAATTTACTA-3′) primers were used for HRM–PCR, which produced a 50-bp product using a denaturation temperature of 55.5 °C. The sequence (SEQ) forward (5′-CAGTGAACATGTGCCCT-3′) and SEQ reverse (5′-CTGCTGATTTCTTTCTAGCA-3′) primers were used for sequencing PCR, which produced a 203-bp product using a denaturation temperature of 52.0 °C. The HRM–PCR was performed using a thermal gradient of 50 to 98 °C in a C-2000 PCR amplifier (Bio-Rad Laboratories, Hercules, CA) before sequencing PCR was performed in a 96-well Light Scanner TMHR-I96 (Idaho Technology, UT). The melting curve was constructed using a slope of 0.3 °C/s. The Light Scanner Call IT software (BioFire Diagnostics, Inc., UT) was used to analyze the melting curve and determine the genotype.

### 2.4 Statistical analysis

All of the statistical analyses were performed using the SPSS, Version19.0, statistical software (IBM, Armonk, NY). The statistical power for our study was estimated to evaluate sample size. A sample size of 250 subjects in each group provided 80% power for detecting the TT genotype, with 75% power for the control group and 85% power for the PCa group based on a 2-sided (α = 5%). A total of at least 500 subjects was required, which indicated n ≥ 250 for each group. Continuous variables, including age and PSA level, are reported as the median and interquartile range (IQR), and the categorical variables are reported as the number of observations and percentage. The rs1058205 genotype frequencies were evaluated to identify significant deviation from Hardy–Weinberg equilibrium. The genotype and allele frequencies for rs1058205 in the PCa and control groups were calculated. Intergroup differences were evaluated using a chi-squared analysis. Logistic regression was used in separate analyses to evaluate the associations between the rs1058205 genotypes and the PCa risk category, overall risk of PCa, and age-adjusted risk of PCa, as well as the corresponding odds ratio (OR) and 95% confidence interval (CI). The level of statistical significance was set at *P* < 0.05. All *P* values were 2-sided.

## 3 RESULTS

### 3.1 Demographic and clinical characteristics

A total of 268 male PCa patients and 298 healthy control subjects were included in our study. The age and clinical characteristics of the participants are shown in Table 1. No significant difference in age was observed between the PCa patients (median: 58 years; IQR: 52–62 years) and the control subjects (median: 62 years; IQR: 53–67 years; *P* > 0.05). The mean PSA level in the PCa group was significantly higher than that in the control group (26.89 ± 15.32 vs 2.62 ± 1.24 ng/mL, respectively; *P* < 0.000). In the PCa group, 101 (37.7%) of the patients had PSA ≥ 20 ng/mL, and 167 (62.3%) of the patients had PSA < 20 ng/mL. A total of 71 (26.5%) of the PCa patients had a Gleason score >7, whereas 197 (37.7%) of them had a Gleason score ≤7. Thirty-two (11.9%) of the PCa patients had malignant PCa, based on Jewett-Whitmore tumor stage C or D, and 236 (88.1%) of them had nonmalignant tumors, based on Jewett-Whitmore stage A or B. The distribution of PCa risk was 38.4% (n = 103) low-risk patients, 49.6% (n = 133) moderate-risk patients, and 11.9% (n = 32) high-risk patients.

**Table 1:**
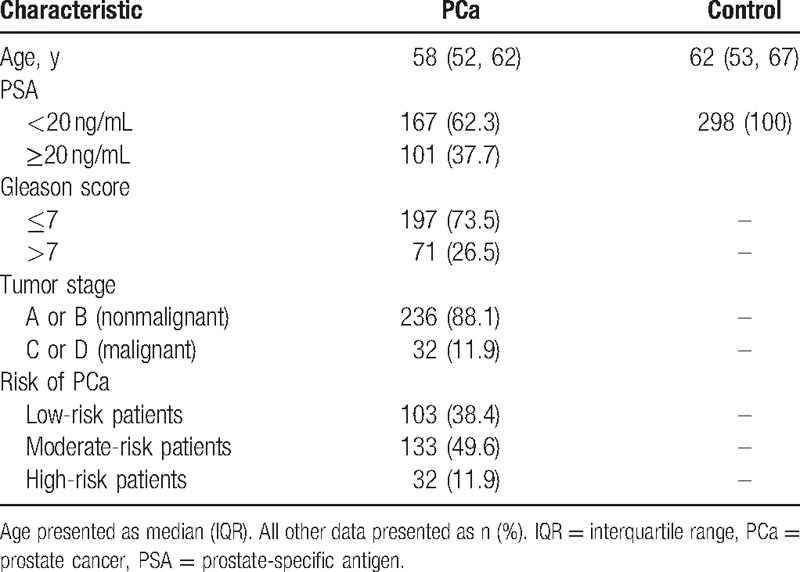
Characteristics of the PCa and control groups.

### 3.2 Genotype distributions

The genotype and allele frequencies for rs1058205 are summarized in Table 2. The genotype distribution did not deviate significantly from the expectations for Hardy–Weinberg equilibrium, with *P* > 0.05 for both groups. The frequencies of the TT, TC, and CC genotypes in the PCa group were 0.78 (n = 209), 0.205 (n = 55), and 0.015 (n = 4), respectively. The frequencies of the TT, TC, and CC genotypes in the PCa group were 0.68 (n = 202), 0.30 (n = 89), and 0.023 (n = 7), respectively. The TC genotype was significantly more prevalent in the control group (*P* = 0.049). No significant differences in the frequencies of the TT or CC genotypes were observed between the PCa and control groups (*P* > 0.05 for both). The T and C allele frequencies in the PCa group were 0.88 and 0.12, respectively, and the T and C allele frequencies in the control group were 0.83 and 0.17, respectively. The genotype and allele distributions suggested that the TC genotype might protect against PCa.

**Table 2:**
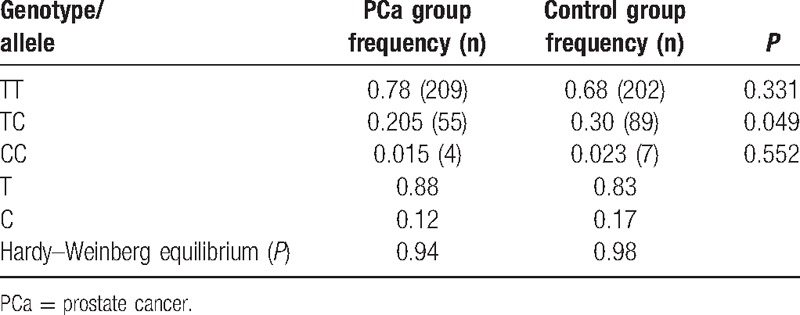
Genotype and allele frequencies for rs1058205.

### 3.3 Differences in PSA level according to rs1058205 genotype

The PSA levels of the participants according to rs1058205 genotype are shown in Table 3. The PSA levels in subjects with the TC genotype were significantly lower than those in subjects with the TT and CC genotypes in both the PCa group and control groups (*P* < 0.010). The PCa patients with the TT genotype had a median PSA level that was 12 fold higher than that in the control subjects with the TT genotype, whereas PCa patients with the TC or CC genotype had a median PSA level that was 9 fold higher than that in their counterparts in the control group. These results suggest that the TT genotype contributed to increased levels of PSA in Chinese PCa patients and that the TC genotype contributed to lower PSA in the PCa group.

**Table 3:**
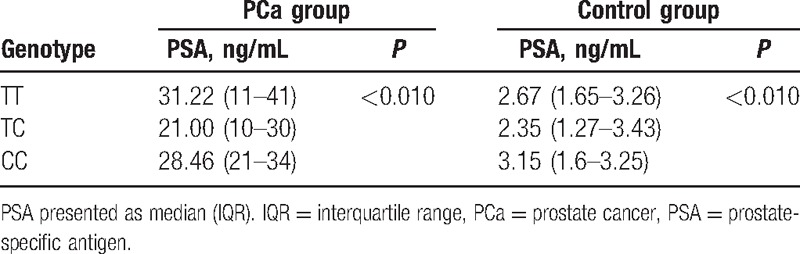
Serum PSA levels according to rs1058205 genotype.

### 3.4 Associations between rs1058205 genotype and the risk of PCa

The rs1058205 genotypes that correlated with higher PSA levels were included in the logistic regression analysis. The results of the logistic regression analysis of rs1058205 genotypes and the overall risk of PCa are shown in Table 4. Using the TC genotype as the reference, the TT genotype was significantly associated with the incidence of PCa (OR: 1.674; 95% CI: 1.136–2.467; *P* < 0.010), whereas the CC genotype was not (*P* = 0.900). Adjustment for age did not alter these associations substantially. The distributions of the TT and TC genotypes in the PCa and control groups were stratified based on PCa risk category, and the results of the logistic regression analysis of rs1058205 genotype and PCa risk category are shown in Table 5. The frequency of the TT genotype was significantly higher in the high- and moderate-risk PCa patients, compared with that of the TC genotype in the high- and moderate-risk PCa patients (*P* = 0.027 and 0.007, respectively). Using the TC genotype as the reference, the TT genotype was significantly associated with a moderate PCa risk (OR: 2.000; 95% CI: 1.205–3.322) and high PCa risk (OR: 3.676; 95% CI: 1.080–12.50). These findings suggest that the TT genotype is a risk factor for PCa and that the TC genotype may diminish PCa tumorigenesis, based on the inclusion of the Gleason score and tumor stage in the risk category assignment and the low frequency of the TC genotype in high- and moderate-risk PCa patients.

**Table 4:**
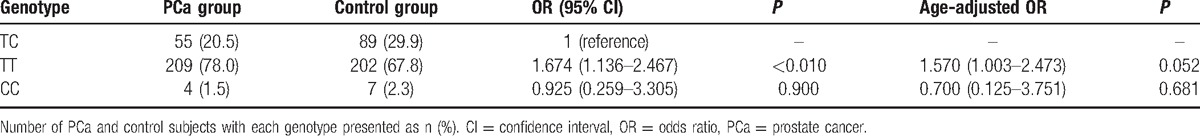
Associations between rs1058205 genotypes and risk of PCa in PCa and control groups.

**Table 5:**
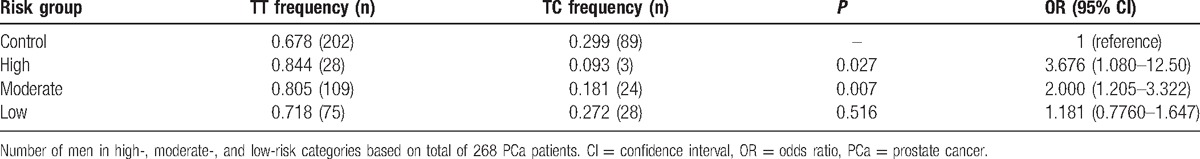
Associations between rs1058205 genotypes and PCa risk category.

## 4 DISCUSSION

In our present study, we found that the frequency of the TC genotype was lower in our PCa patients than that in our healthy control subjects (*P* < 0.05). The PSA level of subjects with the TC genotype of rs1058205 was significantly lower than those of subjects with the TT or CC genotypes in both the PCa and control groups (*P* < 0.010 for both). The TT genotype was associated with a moderate risk and high risk of PCa (*P* = 0.007 and 0.027, respectively). These results suggest that the TT genotype of rs1058205 elevates PSA and increases the risk of PCa in Chinese men. Our results also indicated that the TC genotype might provide protective effects against elevated PSA and PCa pathogenesis in Chinese men.

Our findings are consistent with previous studies, which reported that the TT genotype was associated with higher total serum PSA in Swedish men,^[12]^ Caucasian Americans of European Ancestry,^[20]^ and African-Americans,^[13]^ compared to their respective control group counterparts with the TC or CC genotype. By contrast, Bensen et al^[13]^ also found that the TT genotype of rs1058205 was not associated with elevated PSA in their Caucasian American subjects. Although studies of rs1058205 and PCa in China are scant, in one such study by Zhang et al,^[14]^ an association between the TT genotype and the risk of PCa in Han Chinese men was not observed. However, the C and T allele frequencies they reported for Han Chinese men in Beijing and Tianjin (Northern China) differed from those we observed in our study population in Daqing (Northeast China). Therefore, our findings might not be representative of men in all regions of China. It is also possible that the risk of PCa is influenced by unknown environmental factors, which may vary between different geographic regions.

Previous studies have shown that rs1058205 was associated with lower serum PSA in Swedish and African American men,^[12,13]^ and that the TT genotype of rs1058205 was associated with reduced PCa aggressiveness in Caucasians.^[13]^ Penney et al^[20]^ showed that the C allele of rs1058205 was significantly associated with lower PSA and a reduced risk of PCa in predominantly Caucasian American control subjects and that detection bias resulting from the use of PSA-based screening for clinical diagnosis could not fully explain the association observed between variation at rs1058205 and the risk of PCa. By contrast, the T allele of rs1058205 was not associated with PSA level, PCa incidence, tumor stage, or Gleason score in predominantly Caucasian American PCa patients.^[20]^ The findings of these studies collectively suggest that the biological effect of rs1058205 may contribute to protection against PCa in at least some populations.

The rs1058205 SNP occurs in the 3′ untranslated region of *KLK3* within the 19q13.33 locus. The effect of sequence variation at rs1058205 (T > C) on KLK3 transcription or posttranscriptional RNA processing is unclear, but our data and those of other studies indicate that PSA expression is downregulated in TC heterozygotes. It is possible that the sequence variation in rs1058205 heterozygotes affects the binding of transcription factors or other DNA-binding proteins at the transcriptional unit on 1 chromosome, which might result in the downregulation of KLK3 transcription in trans. Lower PSA is associated with a reduced rate of tumor growth in vitro and lower tumor weight in vivo.^[8,21]^ These previous findings support the correlation between the TC genotype of rs1058205 and reduced serum PSA in both PCa patients and healthy control subjects and the association between the TT genotype and increased PCa risk that were observed in our present study and relevant previous studies by other investigators.^[20]^

A large number of studies have investigated the relationship between PCa and SNPs in genes involved in hormone-receptor signaling, tumor suppression, cell cycle regulation, apoptosis, and extracellular adhesion. To date, genome-wide association studies have identified 77 susceptibility SNP loci for PCa, which have been estimated to contribute approximately 30% to the familial risk of PCa, demonstrating the value of SNP analysis for PCa screening.^[6]^ However, given the large number of susceptibility loci and the relatively small contribution of a single SNP, it is possible that differences in the contributions of loci based on ethnicity are proportionately small, the detection of which may therefore be easily confounded by insufficient sample size or variation in other unassessed risk factors. We assessed multiple clinical variables in our stratified analysis of the effect of rs1058205 on PCa risk, which may have increased the sensitivity of our analysis. However, our study sample was relatively small, which may have diminished the statistical power of our analysis. Similar future studies of larger samples of Han Chinese men are warranted to confirm our findings.

## 5 CONCLUSION

The TT genotype of rs1058205 correlates with elevated PSA and is associated with a moderate-to-high risk of PCa in Han Chinese men. The TT genotype might be a useful biomarker for genetic screening to identify Chinese men at high risk of PCa and as a postoperative prognosticator in Chinese PCa patients. Future studies of the TC genotype of rs1058205 are warranted to clarify whether it exerts protective effects against PCa.
